# PIP2-Effector Protein MPRIP Regulates RNA Polymerase II Condensation and Transcription

**DOI:** 10.3390/biom13030426

**Published:** 2023-02-24

**Authors:** Can Balaban, Martin Sztacho, Ludovica Antiga, Ana Miladinović, Masahiko Harata, Pavel Hozák

**Affiliations:** 1 Department of Biology of the Cell Nucleus, Institute of Molecular Genetics of the Czech Academy of Sciences, 142 20 Prague, Czech Republic; 2 Laboratory of Molecular Biochemistry, Division of Life Science, Graduate School of Agricultural Science, Tohoku University, 468-1, Aramaki Aza Aoba, Aoba-ku, Sendai 980-0845, Japan

**Keywords:** PIP2, RNA polymerase II, transcription, phase separation, MPRIP

## Abstract

The specific post-translational modifications of the C-terminal domain (CTD) of the Rpb1 subunit of RNA polymerase II (RNAPII) correlate with different stages of transcription. The phosphorylation of the Ser5 residues of this domain associates with the initiation condensates, which are formed through liquid-liquid phase separation (LLPS). The subsequent Tyr1 phosphorylation of the CTD peaks at the promoter-proximal region and is involved in the pause-release of RNAPII. By implementing super-resolution microscopy techniques, we previously reported that the nuclear Phosphatidylinositol 4,5-bisphosphate (PIP2) associates with the Ser5-phosphorylated-RNAPII complex and facilitates the RNAPII transcription. In this study, we identified Myosin Phosphatase Rho-Interacting Protein (MPRIP) as a novel regulator of the RNAPII transcription that recruits Tyr1-phosphorylated CTD (Tyr1P-CTD) to nuclear PIP2-containing structures. The depletion of MPRIP increases the number of the initiation condensates, indicating a defect in the transcription. We hypothesize that MPRIP regulates the condensation and transcription through affecting the association of the RNAPII complex with nuclear PIP2-rich structures. The identification of Tyr1P-CTD as an interactor of PIP2 and MPRIP further points to a regulatory role in RNAPII pause-release, where the susceptibility of the transcriptional complex to leave the initiation condensate depends on its association with nuclear PIP2-rich structures. Moreover, the N-terminal domain of MPRIP, which is responsible for the interaction with the Tyr1P-CTD, contains an F-actin binding region that offers an explanation of how nuclear F-actin formations can affect the RNAPII transcription and condensation. Overall, our findings shed light on the role of PIP2 in RNAPII transcription through identifying the F-actin binding protein MPRIP as a transcription regulator and a determinant of the condensation of RNAPII.

## 1. Introduction

The mammalian C-terminal domain (CTD) of the Rpb1 subunit of RNA polymerase II (RNAPII) comprises a 52-heptad repeat, with the consensus sequence of Tyr1-Ser2-Pro3-Thr4-Ser5-Pro6-Ser7 [1]. The differential phosphorylation of this domain correlates with the stages of the RNAPII transcription and coins the term “CTD code” [2]. Throughout transcription, RNAPII interacts with kinases and phosphatases that establish two major regulatory points which limit the rate of the transcription—the initiation (RNAPII recruitment to a promoter), and the pause-release [3].

During initiation, RNAPII is recruited to the promoter region by general transcription factors (TFs) that recognize specific promoter sequences and mediate the assembly of the pre-initiation complex (PIC) [4]. The transcription factor, Nuclear Myosin I (NMI), is an essential component of the initiation complex that is required for the formation of the first phosphodiester bond of the transcript [5]. The RNAPII initiation complex is primarily phosphorylated at its Ser5 residues of the Rpb1¬-CTD, which is mediated by both CDK7 of TFIIH [6] and the Positive Elongation Factor (P-TEFb) [7,8,9]. Upon Ser5 phosphorylation, RNAPII escapes from the promoter and pauses at the promoter-proximal region (+20 to +60 bp) [10]. The pausing of RNAPII provides a vital window to facilitate the integration of multiple cellular signals that determine the transcriptional output [11]. At this stage, RNAPII associates with macromolecular condensates that are formed through liquid-liquid phase separation (LLPS) [6,12]. These condensates are enriched in coactivators such as CDK9, providing a platform to initiate the elongation [6,13]. The Tyr1 phosphorylation of the CTD peaks near the promoter region and controls the pausing of RNAPII, while lower levels of Tyr1 phosphorylations are maintained for efficient termination of the transcription [2,14,15]. The Tyr1 phosphorylation of the CTD at the promoter-proximal region was shown to alter the specificity of P-TEFb from Ser5- to Ser2-CTD [9]. This process is essential to trigger the release of RNAPII, as Ser2 phosphorylation of the CTD abolishes the affinity of RNAPII for the initiation condensate [6]. The released RNAPII proceeds to elongation and relocates toward nuclear speckles where it interacts with the splicing machinery [13,16].

Phosphatidylinositol 4,5-bisphosphate (PIP2) is the most abundant phosphoinositide in the nucleus and it contributes to the nuclear architecture by regulating processes such as chromatin remodeling, DNA-damage response, and gene expression [17]. PIP2 localizes to nuclear speckles, nucleoli, and nucleoplasm, which is determined by electron, light, and super-resolution microscopy [18,19,20,21,22,23]. In nuclear speckles, PIP2 is associated with small nuclear ribonucleoproteins (U1-U6 snRNAs) and the hyper-phosphorylated form of RNAPII [24]. In the nucleolus, PIP2 interacts with many proteins including UBF and fibrillarin, and it participates in the rDNA transcription [21,25,26]. In a previous study, we defined novel nuclear PIP2-containing structures (referred to as Nuclear Lipid Islets, or NLIs) which were observed from 40 to 100 nm foci [21]. The nascent transcripts were visualized at the periphery of NLIs and RNA was shown to be essential for their integrity. These structures were determined to interact with Ser5-phosphorylated CTD and NMI, suggesting a role in the transcription initiation [5,21]. Therefore, we hypothesize that NLIs might serve as scaffolding platforms, which facilitate the formation of the initiation condensates, enabling RNAPII-dependent transcription [21].

Recently, we have identified the PIP2-nuclear interactome and defined the processes that are associated with PIP2-effectors [27]. The Myosin Phosphatase Rho-Interacting Protein (MPRIP) was revealed as a promising candidate protein from the group of actin regulators, and it was identified in the complex with RNAPII and NMI [27,28]. MPRIP contains two PH-domains for PIP2 interaction and it localizes to the nuclear speckles and NLIs. The overexpression experiments showed that GFP-MPRIP forms LLPS condensates that are able to bind nuclear actin fibers [28].

Actin is a transcription regulator that is required for the formation of the pre-initiation complex (PIC) [5]. It binds to the RPABC2 and RPABC3 subunits of all three RNA polymerases and interacts with the CTD of RNAPII [29,30]. Moreover, the activity of the CDK9 depends on actin to trigger the promoter-proximal release by Ser2 phosphorylation of the CTD. After the promoter-proximal release, actin remains bound to the hyper-phosphorylated CTD and interacts with heterogeneous nuclear ribonucleoproteins to regulate transcription elongation, through histone acetylation and mRNA processing [31,32,33,34,35].

The polymerization of nuclear actin disrupts the interaction of monomeric actin with RNAPII, which reduces transcription levels and inhibits cell proliferation [36]. Serum stimulation was shown to induce nuclear actin polymerization, which affects the formation of RNAPII condensates [37,38]. Furthermore, serum stimulation was determined to induce N-WASP and Arp2/3-dependent polymerization of short actin filaments, which facilitate the formation of discrete RNAPII condensates [39]. Similarly, many other actin cytoskeleton regulatory proteins are known to translocate to the nucleus and affect the nuclear actin dynamics. However, the intrinsic mechanisms showing how these proteins regulate nuclear actin-polymerization and consequently, the RNAPII transcription, remains elusive [40].

With this study, we report the evidence of a novel transcription regulator, MPRIP, that determines the association of RNAPII with PIP2. We show that MPRIP regulates transcription through potentiating the association between Tyr1-phosphorylated RNAPII and PIP2-containing nuclear structures. The depletion of MPRIP increases the association of the Ser5-phosphorylated RNAPII with PIP2 and promotes the formation of the condensates. Our data indicate that the depletion of MPRIP impairs the transcription, presumably due to a defect during the pause-release of RNAPII. The F-actin binding capacity of MPRIP further suggests that the nuclear actin polymerization can regulate the RNAPII transcription at pause-release. Overall, our results indicate that MPRIP controls the RNAPII condensation and transcription by regulating the association between the RNAPII and nuclear PIP2.

## 2. Materials and Methods

### 2.1. Cell Cultures and Transfections

Human osteosarcoma (U2OS, ATCC no. HTB96) cells were grown in Dulbecco’s modified Eagle’s medium (DMEM, D6429, Merck, NJ, USA), supplemented with 10% FBS at 37 °C in a humidified 5% CO_2_ atmosphere.

The 5-Fluorouridine (5-FU; F5130, Merck, NJ, USA), which was used for nascent transcript labeling, was initially diluted in water to 100 mM and the aliquots were kept at 20 °C as stock solutions. Then, the stock solutions were diluted in DMEM with 10% FBS to a final concentration of 2 mM. The U2OS cells were grown on cover glasses and were incubated for 20 min in the media with 2 mM 5-FU. After three washes with PBS, the cells were fixed, permeabilized and stained, as described in Section 2.3.

For transfections of the GFP-tagged fragments, Lipofectamine 3000 (L3000015, Invitrogen, MA, USA) was used according to the manufacturer’s protocol. Stable cell lines were established by single cell sorting of the transiently transfected cells, which were further kept in selective media for three weeks with a final concentration of 0.5 mg/mL G418 (G8168, Merck, NJ, USA). The cells were evaluated by western blot and fluorescence light microscopy [28].

MISSION esiRNA (EHU141181, Merck, NJ, USA) was used to perform post-transcriptional silencing of the human MPRIP gene. This product is composed of a pool of hundreds of siRNA (21 bp each) that covers a region of 300–600 bp of the target mRNA. Each individual siRNA has a concentration of around 50 pM in the pool, which significantly lowers the off-target effect. MISSION siRNA Universal Negative Control #1 (SIC001, Merck, Rahway, NJ, USA) was used as the negative control of the MPRIP silencing.

U2OS cells were plated 24 h before transfection at 40% confluency. Cells were transfected using RNAiMax according to the manufacturer’s instructions (13778075, Invitrogen, Waltham, MA, USA). The media was replenished 6 h after transfection and the cells were further incubated for 48 h. Knock-down efficiency was confirmed by western blotting (WB).

### 2.2. Constructs and Antibodies

pDEST53-GFP-MPRIP (Human Isoform 3 of MPRIP) plasmid was used for the over-expression of MPRIP in U2OS cells [28]. The N-terminal (1–450th amino acid) and C-terminal (450–1000th amino acid) regions of MPRIP were amplified by PCR using pDest53-GFP-MPRIP as a template. The N-terminal region was inserted in pEGFP-N1, while the C-terminal region was inserted in pEGFP-C1. For the detailed structure of the fragment, please see Balaban et al., 2021 [28]. The stable cell lines expressing the constructs were examined by confocal microscopy and WB [28].

Primary antibodies (used in concentrations according to the manufacturer’s instructions):

- Anti-MPRIP antibodies: HPA022901 (Sigma, MO, USA) and sc-515720 (Santa Cruz, TX, USA);
- Anti-RNAPII CTD Phospho S5 antibodies: ab5131 and ab5408 (Abcam, Cambridge, UK);
- Anti-RNAPII CTD Phospho Tyr1 antibodies: 61383 and 91219 (Active Motif, CA, USA);
- Anti-RNAPII CTD Phospho S2 antibody: ab5095 (Abcam, Cambridge, UK);
- Anti-BrdU antibody: ab152095 (Abcam, Cambridge, UK);
- Anti-PP1 antibody: HPA046833 (Sigma, MO, USA);
- Anti-PI(4,5)P2 antibody: Z A045, clone 2C11 (Echelon Biosciences Inc., Salt Lake City, UT, USA);
- Anti-beta-Actin antibody: ab8227 (Abcam, Cambridge, UK);
- Anti-MYO1C antibody, detects all isoforms: A, B (Nuclear Myosin I) and C; HPA001768 (Merck, NJ, USA);
- Anti-GFP antibody: ab6556 (Abcam, Cambridge, UK).

Secondary antibodies for immunofluorescence labeling (used at 1:400 dilution):

- Goat anti-Mouse IgM (Heavy chain) Cross-Adsorbed, Alexa Fluor 568, A-21043 (Invitrogen, MA, USA);
- Goat anti-Mouse IgG (H+L) Highly Cross-Adsorbed, Alexa Fluor 488, A-11029 (Invitrogen, MA, USA);
- Goat anti-Mouse IgG (H+L) Highly Cross-Adsorbed, Alexa Fluor 568, A-11031 (Invitrogen, MA, USA);
- Goat anti-Rabbit IgG (H+L) Cross-Adsorbed, Alexa Fluor 568, A-11011 (Invitrogen, MA, USA);
- Goat anti-Rabbit IgG (H+L) Highly Cross-Adsorbed, Alexa Fluor 488, A-11034 (Invitrogen, MA, USA);
- Goat anti-Rat IgG (H+L) Cross-Adsorbed, Alexa Fluor 488, A-11006 (Invitrogen, MA, USA).

Secondary antibodies for WB (used at 1:10,000 dilutions):

- IRDye 680RD Donkey anti-Mouse IgG, 926-68072 (LI-COR Biosciences, NE, USA);
- IRDye 800CW Donkey anti-Mouse IgG, 926-32212 (LI-COR Biosciences, NE, USA);
- IRDye 800CW Donkey anti-Rabbit IgG, 925-32213 (LICOR Biosciences, NE, USA);
- IRDye 680RD Donkey anti-Rabbit IgG, 926-68073 (LI-COR Biosciences, NE, USA);
- IRDye 800CW Goat anti-Rat IgG, 926-32219 (LI-COR Biosciences, NE, USA).

### 2.3. Immunofluorescence Labeling

U2OS cells were grown on high performance cover glasses of 12 mm diameter with restricted thickness-related tolerance (depth = 0.17 mm ± 0.005 mm) and the refractive index = 1.5255 ± 0.0015 (Marienfeld 0107222). After three washes with PBS, the cells were fixed with 2% formaldehyde for 20 min and permeabilized with 0.1% Triton X-100 in PBS for 5 min at room temperature (RT). Nonspecific binding was blocked by 5% bovine serum albumin (BSA) in PBS for 20 min at RT. The cells were incubated for 1 h at RT with the corresponding primary antibodies. After several washes with PBS for 15 min (3 × 5 min) the cells were incubated for 45 min with the secondary antibodies at RT. Then, the cells were washed by another round of 15-min (3 × 5 min) PBS washing. Cells were mounted using 90% glycerol with 4% N Propyl Gallate (NPG). For the super resolution microscopy, the cells were washed with distilled water for 10 min and then dried for 5 min at RT before mounting.

### 2.4. Confocal and Stimulated Emission Depletion (STED) Microscopy

For confocal imaging, a Leica TCS SP8 confocal microscope was employed with a Leica HC PL APO 63x/1.40 oil CS2 objective. The images of one technical repetition were acquired at the same resolution format and zoom value for accurate image analysis.

STED imaging was performed with a Leica TCS SP8 STED 3× microscope, equipped with a Leica DFC365 FX digital camera with a STED white CS 100 × 1.40 NA oil objective for optimized overlay of excitation and a STED beam (Leica Mikrosysteme Vertrieb GmbH, Wetzlar, Germany). Image capturing was performed using the Leica LAS ×64-bit software package. The resolution format of the images is 1024 × 1024 and the corresponding pixel sizes are 18 nm in both x and y. The acquired images were deconvolved using Huygens Professional software version 19.04 (14 September 2021, Scientific Volume Imaging, Hilversum, The Netherlands, http://svi.nl, accessed on 20 January 2023), using the CMLE algorithm, with SNR:07 and 20 iterations.

### 2.5. Image Analysis

The image analysis was carried out using the Coloc2 function of the FIJI software. The Coloc2 function determines the Pearson’s correlation coefficient, Spearman’s rank correlation value, and Manders’ correlation coefficients (M1 and M2) of the signals from the two channels that were analyzed. The significance of each statistical analysis was determined by the paired and two-tailed Student’s *t*-tests. Each data set was normalized to the average and the standard deviation of all three replicates. The randomized images were obtained by 90-degree rotation of one of the two channels [41].

To determine the number of Ser5P-CTD clusters per cell, we have processed the confocal images as described by Cho et al. First, to minimalize the inter-batch variations of the staining densities, the median of the image was filtered (radius 8 pixels) and subtracted from the raw data. Then, the image was smoothed with a 1-pixel radius Gaussian blur, for accurate counting of the clusters. Finally, the find maxima function was applied to count the number of clusters, of which the intensities were above the defined threshold within a cell nucleus. The nuclear outlines were segmented manually [37].

The segmentation of the nuclear PIP2 signals was performed on two-channel confocal images where the nucleolar regions of each cell nucleus were subtracted manually from each channel by the distinctive staining of PIP2 at the nucleolar region [26]. Then, the PIP2 channel was smoothed by Gaussian blur (Sigma 3) to identify the broad areas of the dense signals that relate to PIP2-rich nuclear speckles. This blurred image was further used as a mask for both channels. The region that overlapped with the blurred structures resulted in the image, showing only the broad areas of the nuclear speckles. On the other hand, the subtraction of the blurred structures resulted in the image, showing only the nucleoplasmic PIP2 signal. Finally, the Coloc2 function of the FIJI software was applied to the images to detect the variations in the signal intensities, localizations, and patterns that occur on PIP2-rich nuclear structures.

### 2.6. Pull-Down Assays

Cells were grown in 15cm dishes to 90% confluency. The cells were washed three times with ice-cold PBS and harvested by scraping in 1x lysis buffer (50 mM Tris-HCl, pH 7.2, 150 mM NaCl, 0.1% NP-40). PhosStop (La Roche Ltd., Basel, Switzerland, 4906837001) and cOmplete™ EDTA-free protease inhibitor cocktail (La Roche Ltd., Basel, Switzerland, 05056489001) were added fresh to the lysis buffer on the day of the experiment. The homogenate was incubated 20 min on ice and then sonicated with Soniprep 150 (MSE, London, UK) benchtop sonicator (1 s on, 1 s off for 30 cycles at power 10 microns of amplitude). Sonicated lysate was spun down at 13,000× *g* for 30 min at 4 °C. The supernatant was collected as the total cell extract. Protein concentrations were determined by Pierce™ BCA Protein Assay (Thermo Scientific, MA, USA, 23227) according to the manufacturer’s protocol and determined to be 1.5 mg/mL per sample.

Three types of beads were used in pull-down assays:

- Control beads for PIP2 pull-downs, P-B000 (Echelon Biosciences Inc., UT, USA);
- PI(4,5)P2 covered beads, P-B045A (Echelon Biosciences Inc., UT, USA); and
- Anti-GFP mAb-Magnetic Beads, MAD153-11 (MBL International, MO, USA).

A total of 30 µL of slurry beads was used per condition. The beads were washed three times with 1 mL of ice-cold lysis buffer and then incubated overnight in the total cell extracts. The supernatant was discarded and the beads were washed five times with 1 mL of ice-cold lysis buffer and boiled in 40 µL of 2× Laemmli buffer for 10 min. The beads were spun down and the supernatant loaded into the SDS gel. After trans-blotting, the membranes were blocked with 5% BSA for 30 min at 4 °C. The membranes were washed by PBS Tween 20 for 15 min at 4 °C. The dilutions of the primary antibodies were prepared in 5% BSA/PBS with sodium azide following each manufacturer’s instructions. The incubation with primary antibodies was done at 4 °C with a duration ranging from 4 h to overnight, depending on the antibody. The incubation with the secondary antibodies was 30 min at 4 °C. For scanning the membranes, LiCor Odyssey Infrared Imaging System 9120 (LI-COR Biosciences, Lincoln, NE, USA) was used.

### 2.7. RNA Isolation Procedure

U2OS cells were plated to six-well plates at 70% confluency 24 h before Lipofectamine RNAiMax transfection was performed according to manufacturer’s instructions (13778075, Invitrogen, MA, USA). After 48 h of incubation, cells were washed with ice cold PBS and lysed by TRIzol (BCCF2003, Sigma, MO, USA) directly at the plates. Cells were scraped with disposable cell scrapers (9112400, Sarstedt, Germany) and 250 µL of chloroform was added to the TRIzol/lysate samples. The samples were centrifuged at 10,000 rpm for 5 min at 4 °C and the upper aqueous phase was collected. After the addition of 550 µL of isopropanol to the collected phase, samples were mixed and centrifuged at 14,000 rpm for 30 min at 4 °C. The pellets were washed with 75% ethanol in DNAse/RNAse-free water and centrifuged at 9500 rpm for 5 min at 4 °C. The pellets were air-dried and resuspended in DNase/RNase-free water. The RNA was then treated with 5 U of DNase I per reaction (E0110-D1, Bioresearch Technologies, Hoddesdon, UK) in a mixture containing 5× DNase reaction buffer (29360, Bioresearch Technologies, Hoddesdon, UK) and 20 U RNase inhibitor (S17857, Applied Biosystem, MA, USA). The samples were incubated at 37 °C for 60 min, followed by DNase deactivation at 75 °C for 10 min.

### 2.8. Reverse Transcription of RNA

Reverse transcription was performed using 1 µg of RNA. The mixture contained: 5× first strand buffer (2105651, Invitrogen, MA, USA), DTT (0.1M; 2385398, Invitrogen, MA USA), oligo dT primers (5 μM; S03475, Applied Biosystem, MA, USA), random hexamers (5 μM; S02218, Applied Biosystem, MA, USA), ultrapure BSA (10 mg/mL; 1504057, Invitrogen, MA, USA), dNTPS (SLBG4980V, Sigma, MO, USA), RNA inhibitor (S17857, Applied Biosystem, MA, USA), and 400 U of reverse transcriptase per reaction (SuperScript III, 1701623, Invitrogen, MA, USA). Reverse transcription was performed under the following reaction conditions: 25 °C for 5 min, 42 °C for 30 min, and then 85 °C for 5 min.

### 2.9. Real-Time Quantitative PCR

Real-time quantitative PCR was performed in three technical replicates for three biological samples following the conditions: 95 °C for 10 s, 95 °C for 15 s, 58 °C for 15 s, and 72 °C for 25 s for 50 cycles with LightCycler 480 SYBR Green I Master (04887352001, Roche, Switzerland). The normalized expression values were determined by siMPRIP/– (siNC), the fold change was calculated by siNC (Avg ct)–siMPRIP (Avg ct), and plotted to show changes in gene expressions using specific primers listed in Table 1. The significance of each statistical analysis was determined by the paired and two-tailed Student’s *t*-tests.

## 3. Results

### 3.1. Nuclear MPRIP Localization Correlates with the Phosphorylated Forms of RNAPII

MPRIP localizes to the mammalian cell nucleus [28]. We have determined MPRIP in the complex with active-RNAPII and NMI, which suggests a role for MPRIP in the transcription process [28]. To clarify the nuclear localization of MPRIP, we visualized endogenous MPRIP in respect to three different phosphorylated forms of RNAPII CTD, which mark the initiation (Ser5P), the transition to elongation (also called initiation-release: Tyr1P), and the elongation (Ser2P) stages of the transcription [2,9,42].

**Figure 1:**
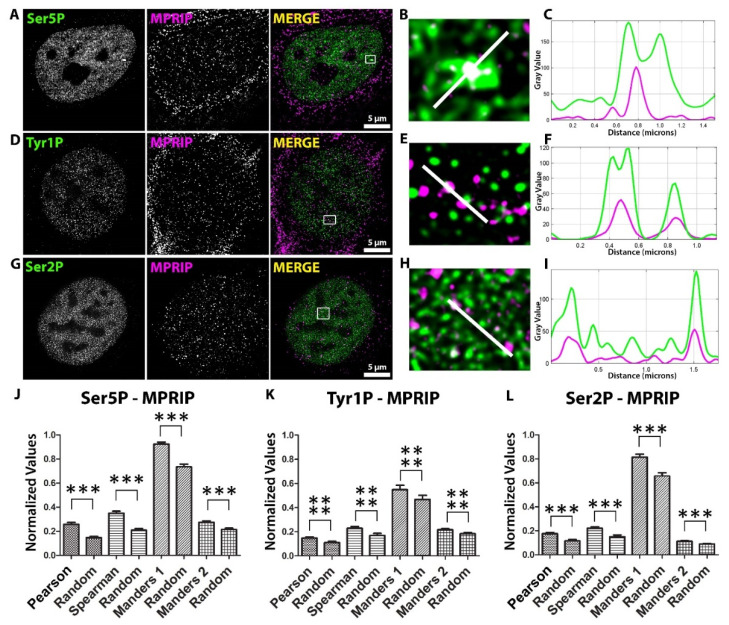
Mutual distribution of MPRIP and phosphorylated forms of RNAPII CTD in the cell nucleus. (**A**,**D**,**G**) Immunofluorescence labeling experiment shows the localization of endogenous MPRIP with Ser5, Tyr1 and Ser2 phosphorylated forms of RNAPII-CTD in the U2OS nucleus. Scale bars represent 5 µm. (**B**,**E**,**H**) Zoomed views of the regions that are marked by a white rectangle in the “merge” images. (**C**,**F**,**I**) The plots represent the intensities of the regions covered by the white lines on the zoomed views. (**J**,**K**,**L**) The column charts show the statistical coefficients of Pearson’s, Spearman’s, and Manders’ tests of MPRIP and phosphorylated RNAPII signal. Manders 1 analysis: MPRIP over RNAPII channel; Manders 2 analysis: RNAPII over MPRIP channel. Columns, marked as ’Random’, were obtained by analyzing randomized images (see M.M.). The triple asterisks “***”correspond to a significance level of *p* ≤ 0.001 and the quadruple asterisks “****” correspond to a significance level of *p* ≤ 0.0001. n = 3, 15 cells/condition for each immunofluorescence experiment.

We performed three indirect immunofluorescence (IF) labeling experiments on U2OS cells with antibodies against MPRIP, Ser5P-, Tyr1P-, and Ser2P-specific RNAPII CTD (Figure 1A–I). We visualized the signals by Stimulated Emission Depletion Microscopy (STED). The spatial distributions of nuclear MPRIP and RNAPII forms were statistically evaluated. The analysis determined significant colocalization and correlation between MPRIP and all of the forms of RNAPII (Figure 1J–L). The nuclear co-distributions of MPRIP showed the highest significance with the Tyr1-phosphorylated form of RNAPII. This indicates that the promotor-proximal pause, which is marked by Tyr1P, represents the stage where MPRIP might be involved.

### 3.2. MPRIP Regulates Transcriptional Output and the Number of the RNAPII Condensates

**Figure 2:**
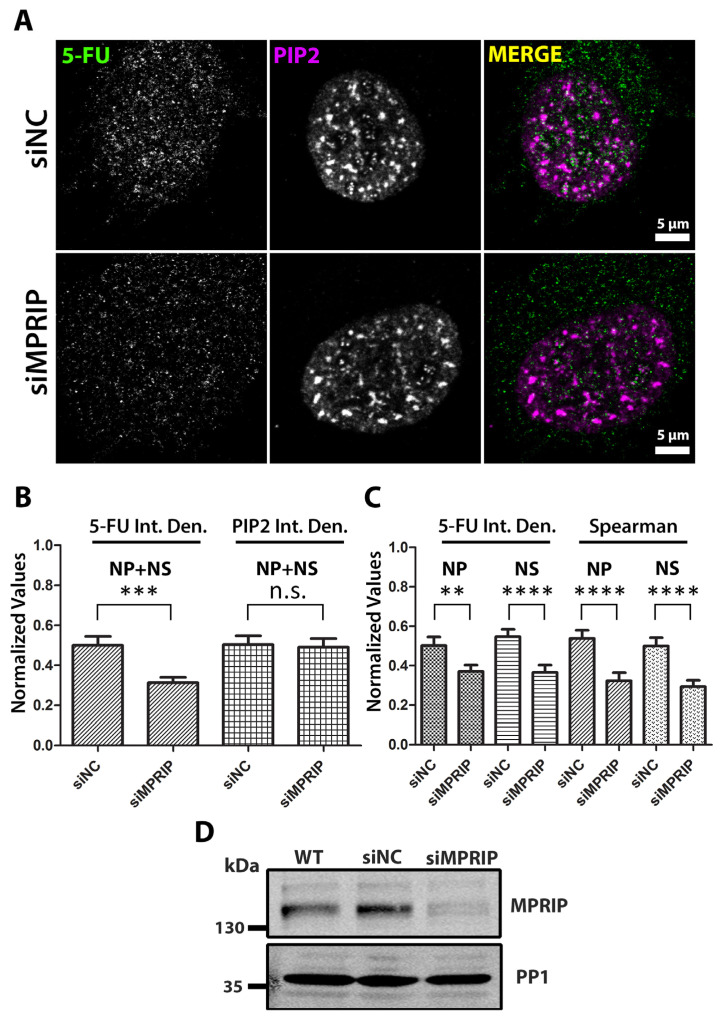
Nascent RNA labeling shows decreased transcriptional output in MPRIP depleted cells. (**A**) Indirect immunofluorescence experiment on U2OS cells showing the 5-FU and PIP2 labeling in control (**siNC**—small interfering Negative Control RNAs) and MPRIP depleted (**siMPRIP**—small interfering MPRIP RNAs) nuclei. Scale bars represent 5 µm. (**B**) The integrated densities (**Int. Den.**) of 5-FU and PIP2 signals in siNC and siMPRIP nuclei are normalized and represented as a column chart. (**C**) The Spearman’s coefficients and the integrated densities of 5-FU signals are analyzed at different PIP2-rich nuclear compartments and the normalized values are shown in the column chart. (**D**) The total cell extracts of Wild-Type (**WT**), siNC and siMPRIP were analyzed by WB to confirm the depletion of MPRIP in U2OS cells. The membranes are blotted with anti-MPRIP specific antibody and with anti-PP1 specific antibody as a loading control. n = 3. **NP**—Nucleoplasmic, **NS**—Nuclear Speckles. **n.s.**—Not significant. The double asterisks “**” correspond to a significance level of *p* ≤ 0.01, the triple asterisks “***” correspond to a significance level of *p* ≤ 0.001, and the quadruple asterisks “****” correspond to a significance level of *p* ≤ 0.0001. n = 3, 10 cells/condition.

The defects during the promoter-proximal pause of RNAPII are associated with a decreased transcriptional output [43]. If MPRIP is involved in the regulation of RNAPII transcription, we would expect a change in the transcriptional output when the cells are MPRIP-depleted. Therefore, we compared the levels of the nascent RNAs in control and MPRIP-depleted cells. We used siRNA-mediated depletion of the endogenous MPRIP, and determined the signal of the incorporated 5-Fluorouridine (5-FU) in nascent RNA transcripts [44,45]. This method enabled us to visualize the location and the amount of nascent RNAs and quantify the variation caused by MPRIP depletion (Figure 2). We found lowered 5-FU signal in MPRIP-depleted cells. These results were supported by the quantitative real-time PCR measurements, which showed decreased mRNA levels of selected housekeeping genes products (HPRT, β2-MG, ALAS, GAPDH, ACTB, 7SK, WDR55, VCL, L3MBT) upon MPRIP depletion in U2OS cells (Supplementary Figure S1). The level of the PIP2 signal remained unchanged in MPRIP-depleted cells compared to the control cells (Figure 2B).

We have previously shown that MPRIP binds specifically to PIP2 in vitro and localizes to nuclear PIP2-rich structures in the cell nucleus: Nuclear Speckles and Nuclear Lipid Islets (NLIs) [28]. Both of these structures are involved in different stages of RNAPII transcription. The NLIs associate with the initiation of the RNAPII transcription, whereas the nuclear speckles associate with the elongation stage [21,46]. We determined a significant decrease in the 5-FU signal in the nucleoplasm and at the nuclear speckles (Figure 2C). As nucleoplasm contains the NLIs, upon MPRIP depletion, the transcription is affected from the initiation stage. Furthermore, the Spearman’s coefficient determines the level of correlation between PIP2 and 5-FU signals and thus defines the strength of the relation of their spatial co-distribution. The depletion of MPRIP leads to a significant decrease of Spearman’s coefficients, irrespective of the intensity of evaluated signals, suggesting that MPRIP depletion mainly affects the 5-FU incorporation in proximity to PIP2. These data underline the importance of PIP2 in RNAPII transcription.

**Figure 3:**
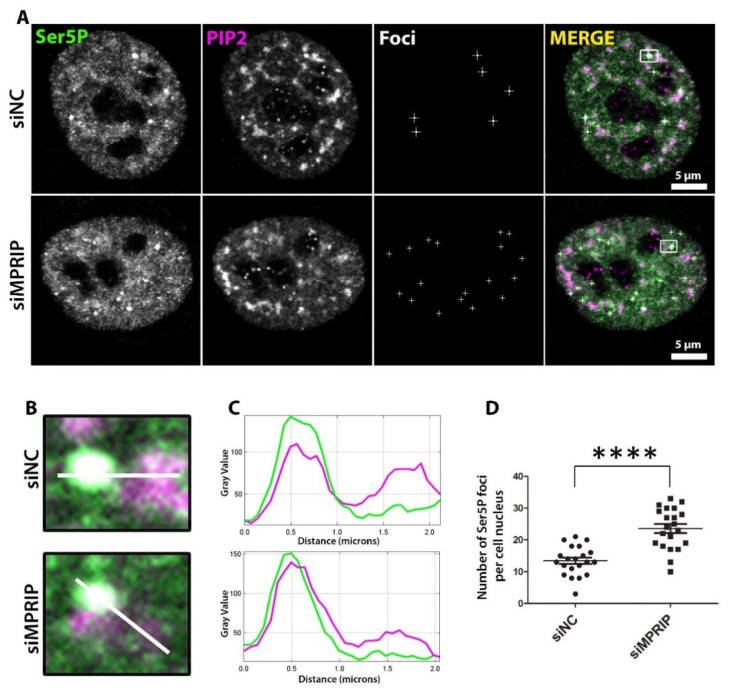
The number of Ser5P-CTD foci increase upon MPRIP depletion in cell nuclei. (**A**) Immunofluorescence labeling experiment on U2OS cells showing the Ser5P-CTD foci in control (**siNC**—small interfering Negative Control RNAs) and MPRIP-depleted (**siMPRIP**—small interfering MPRIP RNAs) nuclei. Scale bars represent 5 µm. (**B**) Zoomed views of foci that are marked by a white rectangle in the “Merge” images. The white lines on the zoomed views represent the regions for the intensity plots on (**C**) where the upper plot corresponds to the intensities of siNC and lower, to siMPRIP. (**D**) The dot plot shows the number of Ser5P-CTD foci per cell nucleus of siNC and siMPRIP. The quadruple asterisks “****” correspond to a significance level of *p* ≤ 0.0001. n = 3.

We have previously proposed that NLIs associate with the initiation stage of the RNAPII transcription [21]. During the initiation stage, the RNAPII-CTD is phosphorylated at Ser5 residues and this specific phosphorylation marks the macromolecular-initiation-condensates that are formed through liquid-liquid phase separation (LLPS) [13,47]. Several studies suggest that the defects in the promoter-proximal pause of transcription inhibit new transcription initiation with an accumulation of Ser5P-CTD at the promoter-proximal region of the gene [10,48,49,50]. Therefore, we investigated the phenotype of the transcription-initiation marker, the Ser5P-CTD, in MPRIP-depleted cells. The confocal microscopy reveals that the number of Ser5P-CTD foci, which represent the initiation condensates [12,50], is significantly increased upon MRPIP depletion (Figure 3). Therefore, these results indicate that MPRIP affects the formation and/or dissolution of the RNAPII initiation condensates. Moreover, if MPRIP depletion is indeed causing a defect at the pause-release of RNAPII, the increased number of condensates might also provide an explanation for the decreased transcriptional output (Figure 2 and Figure 3).

### 3.3. MPRIP Affects the Association of RNAPII with PIP2

**Figure 4:**
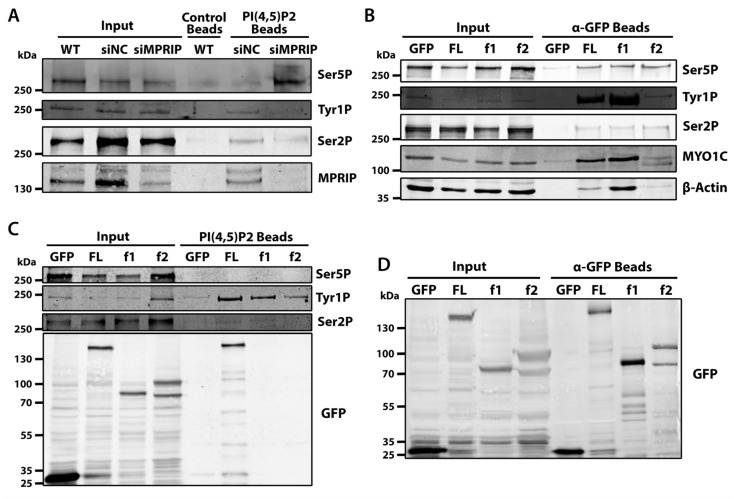
(**A**) **MPRIP regulates the association between RNAPII and PIP2.** PIP2 coated agarose beads are used as bait to pull down interacting proteins from U2OS cell lysates. The membrane is blotted with anti-Ser5P, Tyr1P, Ser2P RNAPII-CTD, and anti-MPRIP antibodies. Empty agarose beads (control beads) are used as a control for unspecific binding. Input is 1% of the cell lysate used for each pull-down experiment. **WT**—Wild Type U2OS cells, **siNC**—small interfering RNAs for Negative Control, **siMPRIP**—small interfering RNAs for MPRIP depletion. n = 3 repetitions. (**B**) **GFP-trap experiment showing the interacting partners of each fragment of MPRIP.** Anti-GFP coated magnetic beads are used as bait to pull down interacting proteins from U2OS cell lysates overexpressing only GFP, GFP-tagged full length (FL) MPRIP, and GFP-tagged N- and C-terminal MPRIP fragments (f1 and f2, respectively). The membrane is blotted with anti-Ser5P, Tyr1P, Ser2P-CTD specific RNAPII, anti-MYO1C, and β-actin antibodies. GFP-only expressing cells are used as a control for unspecific binding. Input is 1%. n = 3. (**C**) **MPRIP promotes the association between Tyr1 phosphorylated CTD and PIP2.** PIP2-coated agarose beads were used as a bait to pull down interacting proteins from U2OS cell lysates that were overexpressing GFP, GFP-tagged full-length (**FL**) MPRIP, and GFP-tagged N- and C-terminal MPRIP fragments (**f1** and **f2**, respectively). The membranes are blotted with anti-Ser5P, Tyr1P, Ser2P-CTD specific RNAPII, and anti-GFP antibodies. GFP-only expressing cells are used as a control for unspecific interactions of GFP-tagged overexpressing proteins. Input is 1%. n = 3. (**D**) **Control of the GFP-trap experiment in** (**B**). Anti-GFP coated magnetic beads are used as bait to pull down interacting proteins from U2OS cell lysates overexpressing GFP-tagged MPRIP protein variants. The membranes are blotted with anti-GFP antibodies. Input is 1% of the cell lysate used for each pull-down experiment. n = 3.

The microscopy experiments indicate that the MPRIP depletion has an impact on 5-FU incorporation at NLIs and nuclear speckles (Figure 2). We have previously shown that the RNAPII associates with PIP2 [21]. Therefore, we sought to assess whether MPRIP affects the association between RNAPII with PIP2 in different stages of transcription. To this end, we determined the association of Ser5P-, Tyr1P-, and Ser2P-CTD with PIP2-conjugated beads in pull-down experiments (Figure 4A). The Ser5P-CTD shows increased association with PIP2 upon MPRIP depletion (Figure 4A). This suggests that the increased number of Ser5P-CTD foci, upon MPRIP depletion (Figure 3), might be formed due to the increased association between RNAPII and PIP2. This is in accordance with the proposed role of PIP2 in the transcription initiation of RNAPII [21]. In MPRIP-depleted cells, the markers for the later stages of RNAPII transcription (Tyr1P and Ser2P) are lower compared to control cells in the pull-downs with PIP2 beads. This indicates that the stages of RNAPII transcription, which follow the initiation, are abrogated by the depletion of MPRIP (Figure 4A). In addition, the overall levels of the CTD phosphorylations do not change upon MPRIP depletion, indicating that MPRIP solely affects the association of RNAPII with PIP2.

We previously identified MPRIP as an interactor of the RNAPII and NMI [28]. Here, we showed the spatial co-distribution of MPRIP with three different phosphorylated forms of RNAPII-CTD (Figure 1). Therefore, to define the stage of RNAPII transcription that involves MPRIP, we sought to determine the predominant RNAPII form that associates with MPRIP and the region of MPRIP that is responsible for this association. In order to do so, we utilized the GFP-tagged MPRIP constructs coding for full-length (FL), N-terminal region (f1: comprising two PH domains and an actin-binding region), and C- terminal region (f2: comprising an intrinsically disordered region which promotes LLPS) [28]. The Tyr1P-CTD reveals as the predominant phosphorylated form that associates with the N-terminal region of MPRIP (Figure 4B). Furthermore, the transcription factor MYO1C (Isoform B corresponds to NMI) and actin, which associate with the initiation of transcription, are also detected in the pull-down with the N-terminal region of MPRIP. These results suggest that MPRIP possesses the ability to bind to RNAPII complex, and affect the association of RNAPII with PIP2, presumably regulating the initiation-release.

Recent data suggest that Tyr1 phosphorylation of CTD marks the promoter-proximal pause of RNAPII [9,14,42]. Our data show that MPRIP affects the number of Ser5P-CTD foci (Figure 3), the transcriptional output (Figure 2), and the association of RNAPII with PIP2 (Figure 1 and Figure 4A). The identification of Tyr1P-CTD as the predominant interactor of MPRIP (Figure 4B) prompted us to elucidate whether MPRIP affects the association between Tyr1P-CTD and PIP2. In order to address this, we overexpressed GFP-MPRIP constructs in U2OS cells and performed pull-down experiments to detect if any phosphorylated form of RNAPII-CTD shows a change in its association with PIP2. The result in Figure 4C shows that the full-length MPRIP promotes the association between the Tyr1P-CTD and PIP2. Yet, it is noteworthy that f1 and f2 mutants also increase the association between Tyr1P-CTD and PIP2, even though they are not pulled-down by PIP2 beads. It is possible that these mutants are interacting with the endogenous MPRIP. Nonetheless, this experiment shows that MPRIP promotes the association between Tyr1P-CTD and PIP2.

**Figure 5:**
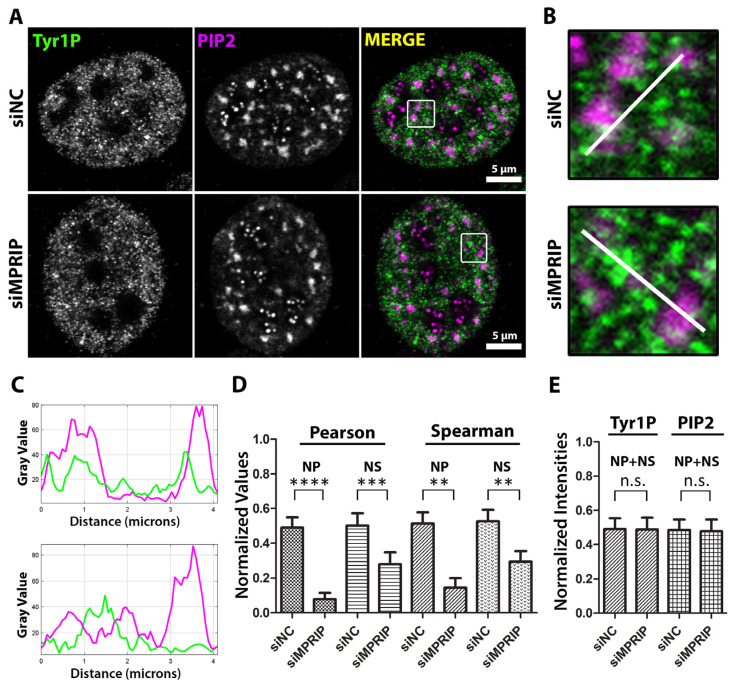
MPRIP depletion leads to the decreased association of Tyr1 phosphorylated RNAPII to PIP2. (**A**) The immunofluorescence experiment visualized by a confocal microscope shows the mutual distribution of PIP2 and Tyr1P-CTD in control and MPRIP depleted U2OS nucleus. Scale bars represent 5 µm. (**B**) Zoomed views of the regions that are marked by a white rectangle in the “merge” images. The white lines on the zoomed views represent the regions of the intensity plots (**C**) where the upper plot corresponds to the intensities of siNC and the lower, to siMPRIP. (**D**) The column chart shows normalized values of Spearman’s and Pearson’s coefficients that are observed in different PIP2-rich nuclear compartments. (**E**) The integrated densities of Tyr1P-CTD and PIP2 signals are normalized and represented as a column chart in control (**siNC**—small interfering Negative Control RNAs) and MPRIP depleted (**siMPRIP**—small interfering MPRIP RNAs) conditions. **NP**—Nucleoplasmic, **NS**—Nuclear Speckles. **n.s.**—Not significant. The double asterisks “**” correspond to a significance level of *p* ≤ 0.01, the triple asterisks “***” correspond to a significance level of *p* ≤ 0.001, and the quadruple asterisks “****” correspond to a significance level of *p* ≤ 0.0001. n = 2, 10 cells/condition.

So far, our results indicated that MPRIP affects the association between PIP2 and RNAPII in vitro. We further tested whether MPRIP depletion affects the localization of Tyr1P-CTD compared to the nuclear PIP2-containing structures (Figure 5). The Pearson’s and Spearman’s analyses of the microscopy images show decreased colocalization and co-distribution between Tyr1P-CTD and nuclear PIP2-containing structures upon MPRIP depletion (Figure 5D). This effect is more prominent at NLIs compared to the nuclear speckles, pointing out that the transcription might be altered from the initiation stage. In accordance with our in vitro data, the signal intensity levels of Tyr1P-CTD and PIP2 remain unchanged upon MPRIP depletion (Figure 5E). Overall, our results confirm the importance of the MPRIP-dependent regulatory axis that determines the association of Tyr1P-CTD with PIP2 as an important factor in defining the transcription levels of RNAPII.

## 4. Discussion

We have previously reported that MPRIP is not only a cytoplasmic protein that regulates actin stress fibers but also an interactor of the RNAPII, localizing to PIP2-containing nuclear structures [27,28]. The overexpression of GFP-MPRIP showed the ability of this protein to form LLPS condensates in the nucleus with the capacity to bind nuclear actin filaments [28]. The growing body of evidence over the past decade emphasizes the importance of nuclear actin regulators in the transcription process [40,51,52]. However, comprehensive knowledge about the details of particular molecular mechanisms remains vastly elusive. Here, we uncover the basis of the molecular mechanism of nuclear MPRIP that affects the RNAPII-mediated transcription and its association with the nuclear PIP2.

Phosphorylation of RNAPII is a tightly regulated process with a number of effects on transcription and subsequent processes [13,53]. Several studies have mapped the CTD phosphorylations during RNAPII activity and identified a correlation with different stages of transcription [2]. We observe that the nuclear MPRIP associates with the subpopulations of three phosphorylated forms of RNAPII CTD (Figure 1). These forms of RNAPII are associated with initiation (Ser5P), pause-release (Tyr1P), and elongation (Ser2P) stages of the transcription [2,9,42]. The statistical analysis of the super-resolution microscopy images revealed that from all three phosphorylated forms of CTD, the Tyr1P-CTD had the most significant association with MPRIP (Figure 1J–L). This suggests that MPRIP might have a role during the pause-release of RNAPII that is marked by Tyr1 phosphorylation of the CTD.

The pause-release of RNAPII is a critical regulatory stage in transcription that determines the transcriptional output [3,9]. The studies show that the defects during the pausing of RNAPII inhibit new transcription initiation, leading to decreased levels of transcription [43,48,49]. Similarly, the MPRIP-depleted cells show lower transcriptional output, indicating a defect in the transcription machinery (Figure 2). Moreover, the Spearman’s coefficients, which determine the spatial correlation between PIP2 and nascent RNA transcripts, show decreased values upon depletion of MPRIP (Figure 2). These results suggest that MPRIP-dependent transcription is linked to the NLIs and nuclear speckles. Indeed, both of these structures are involved in active RNAPII transcription [21,54,55,56]. Considering NLIs are associated with the initiation of transcription [21], the decreased Spearman’s values at the nucleoplasmic region indicate that the transcription might be affected from the initiation stage when MPRIP is depleted.

We previously showed that MPRIP associates with NLIs and the initiation factor, NMI [5,21,28]. The initiation of transcription is characterized by the increasing amounts of Ser5P-CTD that associate with the macromolecular condensates with liquid-like properties [12,50,57]. Several studies have documented the accumulation of Ser5P-CTD at the promoter-proximal region due to the lack of factors that are involved in pause-release [9,43,48,49]. Similarly, we have determined that the MPRIP depletion increases the number of the Ser5P-CTD foci, indicating a defect in the pause-release of RNAPII (Figure 3). Thus, it seems possible that MPRIP interacts with RNAPII in condensates, promoting the release of the transcription machinery from the initiation condensate to enable the transcription elongation.

Factors such as the negative elongation factor (NELF), DRB sensitivity-inducing factor (DSIF), and polymerase-associated factor 1 (Paf1) are known to govern the pausing of the RNAPII, while its release requires the kinase activity P-TEFb [58,59,60,61]. Our results show that MPRIP depletion abolishes the association of Tyr1P-CTD to PIP2 (Figure 4A). This outcome suggests that MPRIP might regulate the pause-release of RNAPII through affecting the association of RNAPII with PIP2 (Figure 4A). In addition, this experiment reveals the increased association between Ser5P-CTD and PIP2 in MPRIP-depleted cells. This might provide an explanation for the increased number of Ser5P-CTD foci in MPRIP depletion, where the increased PIP2 association of Ser5P-CTD might reduce the dissolution of the initiation condensate (Figure 3 and Figure 4A). Taken together, we propose that MPRIP is necessary for the release of RNAPII from initiation condensate by reducing the association of Ser5P-CTD to PIP2. Thus, it is possible that the association between RNAPII and PIP2 determines the transcriptional output through regulating the pausing of RNAPII.

**Figure 6:**
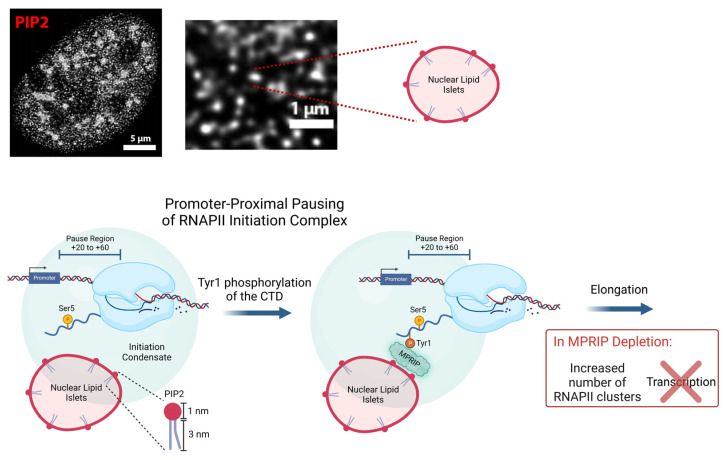
**Model depicting proposed mechanism for the MPRIP-mediated PIP2 association with Tyr1-phosphorylated-RNAPII complex facilitating the release of RNAPII from the promoter-proximal pausing**. The Ser5-phosphorylated RNAPII pauses at the promoter-proximal region (+20 to +60 bp). PIP2-containing nuclear lipid islets (NLIs) associate with the initiation condensate [18,19,20,21]. Following the Tyr1 phosphorylation of the Rpb1 CTD, MPRIP mediates the interaction between the RNAPII complex and NLIs. By regulating the association of the RNAPII complex with PIP2, MPRIP affects the release of the RNAPII complex from the initiation condensate. In case of MPRIP depletion, there is an increased association between Ser5P-CTD and PIP2. We suggest that the change in this association causes a defect in promoter-proximal pause and prevents RNAPII being released from the initiation condensate. As a result, the transcription is downregulated and the number of RNAPII foci is increased.

Studies have shown that Tyr1P-CTD levels peak at the promoter-proximal region, while lower levels of Tyr1P-CTD are maintained for efficient termination of the transcription [2,14,15,42]. Our results reveal that Tyr1P-CTD is the predominant form of RNAPII that associates with the N-terminal region of MPRIP, together with MYO1C (an isoform of NMI) and actin. Considering NMI and actin are components of the initiation complex [5,62,63], this experiment supports our previous indications and postulates that MPRIP acts on the Tyr1P-CTD at the promoter-proximal pausing. Furthermore, we show that the association between Tyr1P-CTD and PIP2 is promoted by GFP-MPRIP overexpression (Figure 4C). This observation is complemented by our microscopy experiments, showing a decrease in Tyr1P-CTD colocalization with both NLIs and nuclear speckles in MPRIP depletion (Figure 5). Both these results, in Figure 4C and Figure 5, suggest that MPRIP might be mediating the association between PIP2-rich structures (NLIs and nuclear speckles) and Tyr1P-CTD. Altogether, our results support the proposition that MPRIP is a factor regulating the pause-release, where it defines the susceptibility of RNAPII to leave the initiation condensate, presumably by mediating the interaction between the RNAPII complex and NLIs (Figure 6).

Interestingly, in contrast to full-length MPRIP, we observe a lack of interaction between the PH-domain-containing N-terminal region of MPRIP and PIP2 (Figure 4C). Similar properties of PH-domains have been observed in a recent study, which demonstrated that isolated PH-domains lose their affinity to PIPs in comparison to their full-length counterparts [64]. Alternatively, it is possible that the conformation of the mutant might favor the interaction with actin over PIP2 (Figure 4C). Since PIP2 and F-actin binding regions of MPRIP overlap [65], the binding to F-actin might hide the PH-domains of the mutant protein, hindering its interaction with PIP2.

The nuclear actin concentration is an important regulator of the RNAPII transcription [66]. The polymerization of nuclear actin can be induced upon serum-stimulation and affects the nuclear actin concentration, through incorporating the monomeric actin into filaments [38,67,68]. The fluctuations in the concentration of an initiation factor such as actin can determine the transcription activity. Indeed, studies have documented that the formation of F-actin can both activate and repress the expression of different genes [52,69]. For example, persistent nuclear F-actin was shown to disrupt actin–RNAPII interaction and impaired RNAPII localization, reducing transcription levels and cell proliferation [36]. In addition, through the activity of actin-binding proteins (N-WASP and Arp2/3), the serum-induced nuclear actin polymerization was shown to facilitate the formation of discrete RNAPII foci [39]. Since MPRIP is a negative F-actin regulator which is able to bind nuclear F-actin [28], it is tempting to speculate that the nuclear F-actin structures regulate RNAPII transcription through MPRIP. However, further research is required to elucidate the exact role of actin underlying the regulatory axis of PIP2 and MPRIP-associated RNAPII machinery.

In summary, we showed that MPRIP represents a novel regulator of RNAPII that affects the number of initiation condensates and thus transcriptional output. We propose that nuclear PIP2 at the surface of NLIs serves as a determinant of RNAPII condensation level in the initiation stage (Figure 6). This was manifested by the changes of RNAPII affinity to PIP2-containing structures in vitro upon MPRIP depletion. In such a scenario, the MPRIP protein promotes the release of RNAPII into the elongation stage through association with Tyr1P-CTD by an as-yet-unknown mechanism. Taken together, our findings represent the functional overlap among nuclear architecture defined by PIP2, which regulates the RNAPII condensation through phase separation, resulting in the modulation of transcriptional output.

## Figures and Tables

**Table 1:** Primers used in this study.

Name	Sequence
>NM_000194.3 Homo sapiens hypoxanthine phosphoribosyl transferase 1 (HPRT)	Fwd: 5′ TGACCTTGATTTATTTTGCATACC 3′ Rev: 5′ CGAGCAAGACGTTCAGTCCT 3′
>NM_004048.4 Homo sapiens beta-2-microglobulin (ß2-MG)	Fwd: 5′ GTATGCCTGCCGTGTGAACCATG 3′ Rev: 5′ CAAATGCGGCATCTTCAAACCTCC 3′
>NM_000688.6 Homo sapiens 5’ -aminolevulinate synthase 1 (ALAS)	Fwd: 5′ CCACTGGAAGAGCTGTGTGATGTG 3′ Rev: 5′ GCGATGTACCCTCCAACACAACC 3′
>NR_001445.2 Homo sapiens RNA component of 7SK nuclear ribonucleoprotein (7SK)	Fwd: 5′ ATTGATCGCCACxxXGTTGATT 3′ Rev: 5′ CGGGGAAGGTCGTCCTCTTC 3′
>NM_001256799 Homo sapiens glyceraldehyde-3-phosphate dehydrogenase (GAPDH)	Fwd: 5′ GTCGGAGTCAACGGATTTGG 3′ Rev: 5′ AAAAGCAGCCCTGGTGACC 3′
>NM_001101 Homo sapiens actin beta (ACTB)	Fwd: 5′ AGGCACCAGGGCGTGAT 3′ Rev: 5′ TCGCCCACATAGGAATCCTT 3′
>NM_014000.3 Homo sapiens vinculin (VCL)	Fwd: 5′ GATGAAGCTCGCAAATGGTC 3′ Rev: 5′ TCTGCCTCAGCTACAACACCT 3′
>NM_031488.5 Homo sapiens histone methyl-lysine binding protein 2 (L3MBT)	Fwd: 5′ AGGCACCAGGGCGTGAT 3′ Rev: 5′ TCGCCCACATAGGAATCCTT 3′
>NM_017706.5 Homo sapiens WD repeat domain 55 (WDR55)	Fwd: 5′ GGAAGACATCGTGCTGGAAG 3′ Rev: 5′ TGGCAAGAGTAGGAAAAGACG 3′

## Data Availability

Data is contained within the article.

## Supplementary Materials

The following supporting information can be downloaded at: https://www.mdpi.com/article/10.3390/biom13030426/s1. Figure S1: Quantitative real-time PCR measurements of mRNA levels of selected housekeeping genes products (HPRT, β2-MG, ALAS, GAPDH, ACTB, 7SK, WDR55, VCL, L3MBT) upon MPRIP depletion in U2OS cells. (A) The column charts show comparison of the ct values in siNC (small interfering Negative Control) and siMPRIP (MPRIP depleted by small interfering RNA) in U2OS cells with statistical significance indicated in three independent experiments; “*” corresponds to a significance level of *p* ≤ 0.05, “**” to *p* ≤ 0.01, “***” to *p* ≤ 0.001, and “****”" to *p* ≤ 0.0001. N = 3, n = 3. (B) The column chart shows the fold change decrease of mRNA levels of indicated genes expression induced by siMPRIP depletion. (C) The chart shows the effect of MPRIP depletion of normalized expression levels of indicated genes with statistical significance indicated. “*” corresponds to a significance level of *p* ≤ 0.05. N = 3, n = 3.
