# Supplementary material for: PIP2-Effector Protein MPRIP Regulates RNA Polymerase II Condensation and Transcription
Source: Biomolecules. 2023 Feb 24;13(3):426. doi: 10.3390/biom13030426 (PMC10046169; doi:10.3390/biom13030426)
Supplement: Supplementary file 1 [file biomolecules-13-00426-s001.zip › biomolecules-2206586-supplementary.pdf]

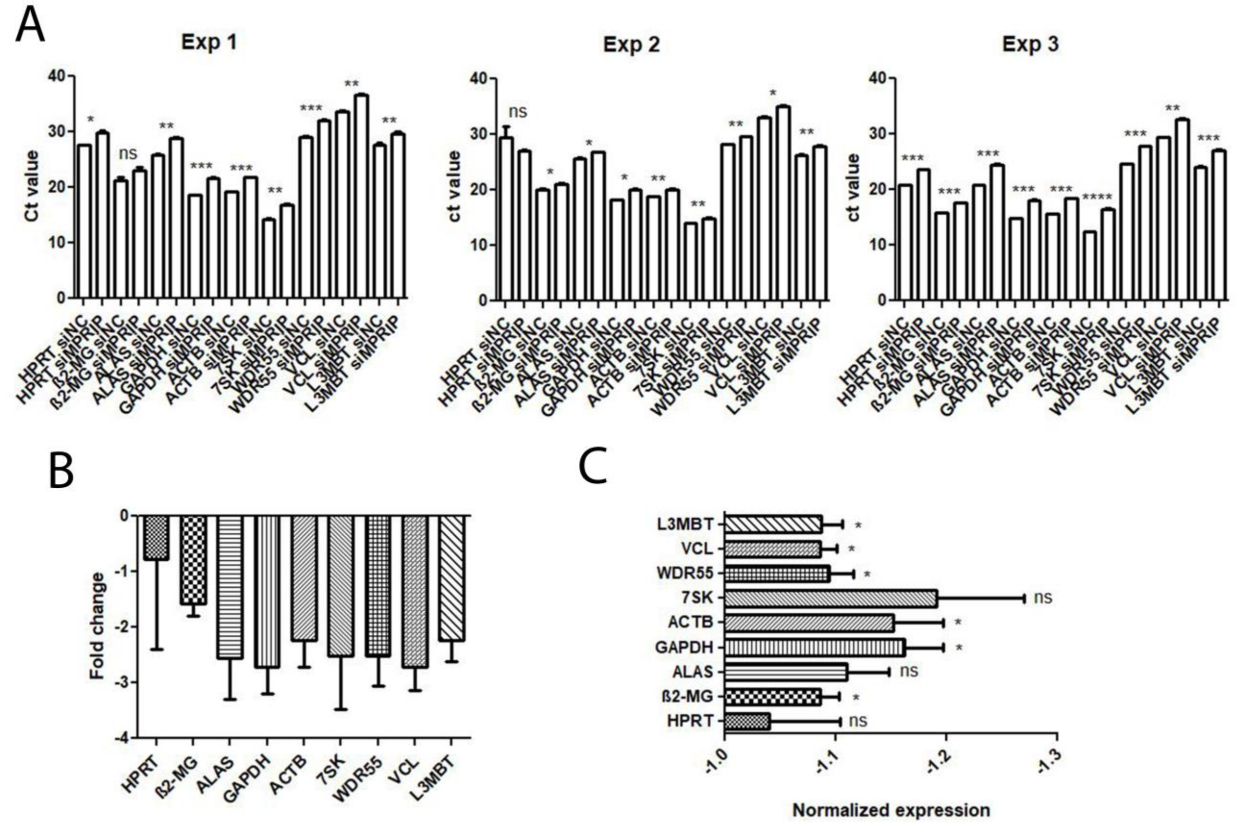

Supplementary Figure S1. Quantitative real-time PCR measurements of mRNA levels of selected housekeeping genes products (HPRT, β2-MG, ALAS, GAPDH, ACTB, 7SK, WDR55, VCL, L3MBT) upon MPRIP depletion in U2OS cells. **(A)** The column charts show comparison of the ct values in siNC (small interfering Negative Control) and siMPRIP (MPRIP depleted by small interfering RNA) in U2OS cells with statistical significance indicated in three independent experiments; "*" corresponds to a significance level of $p \leq 0.05$, "**" to $p \leq 0.01$, "***" to $p \leq 0.001$, and "****" to $p \leq 0.0001$. N= 3, n=3. **(B)** The column chart shows the fold change decrease of mRNA levels of indicated genes expression induced by siMPRIP depletion. **(C)** The chart shows the effect of MPRIP depletion of normalized expression levels of indicated genes with statistical significance indicated. "*" corresponds to a significance level of $p \leq 0.05$. N= 3, n=3.
